# Changes of proapoptotic and antiapoptotic genes affect sensitivity to apoptotic stimuli in impaired contractility due to long term bladder outlet obstruction

**DOI:** 10.1371/journal.pone.0279503

**Published:** 2022-12-27

**Authors:** Jae Heon Kim, Hee Jo Yang, Sung Sik Choi, Hong J. Lee, Yun Seob Song

**Affiliations:** 1 Department of Urology, Soonchunhyang University School of Medicine, Seoul, Republic of Korea; 2 Department of Urology, Soonchunhyang University School of Medicine, Cheonan, Republic of Korea; 3 College of Medicine and Medical Research Institute, Chungbuk National University, Cheongju, Chungbuk, Republic of Korea; 4 Research Institute, e-biogen Inc., Seoul, Republic of Korea; Sao Francisco University: Universidade Sao Francisco, BRAZIL

## Abstract

**Introduction:**

The normal biological process that necessitates cell removal greatly depends on apoptosis. Long term bladder outlet obstruction (BOO) causes damaged smooth muscle cells to undergo apoptosis. However, smooth muscle cell apoptosis that BOO causes is not well known in impaired bladder contractility. Therefore, we designed this study to investigate whether long-term BOO could induce apoptosis activities and to obtain an expression profile of apoptosis related genes.

**Materials and methods:**

We used 10 Sprague-Dawley six-week-old female rats. We separated them equally into two groups: a sham intervention group (group 1) and an eight-week BOO group (group 2). We conducted cystometric evaluation eight weeks following BOO onset, with processing of bladder tissue for PCR array. Every array comprised 84 genes, which were established to contribute to an apoptosis response, cell differentiation and metabolism, and 12 sequences were established for the regulation of loading and the quality of cDNA. We performed real-time PCR. Changes in gene expression presented as a fold increase/decrease. Alterations of more than two-fold constituted the cut-off determining expression.

**Results:**

Group 2 had a greater bladder weight and Impaired bladder contractility. Immunofluorescent staining with CAS3, TUNEL showed increased in the BOO group. In comparison to group 1, group 2 exhibited an at least two-fold upregulation in five genes, the Bcl-2 (15.1), Birc5 (5.8), Cd40lg (7.5), Il10 (16.2), and Naip2 (13.2). They also demonstrated at least a two-fold downregulation in the PRLR (-18.1) gene. Genes Bcl2ald, Circ5, Cd40lg, Il10, Naip2, and PRLR were among the genes with activity against apoptosis. TNF, STAT3 and TP53 mediated the effect that genes had on one another.

**Conclusion:**

This study demonstrated that the relative ratios of pro- and antiapoptotic genes determine bladder cell sensitivity cells to apoptotic stimuli in impaired contractility caused by long term BOO. Although we cannot confirm whether this finding is the result of the decompensated phase of the bladder or the process, the gene expression profiles could explain molecular mechanisms of apoptosis in impaired bladder contractility caused by long-term BOO with further studies.

## Introduction

Bladder outlet obstruction (BOO) is one of the most common condition in elderly [1]. This condition is associated with outflow resistance, which requires high intravesical pressure to overcome, which in turn reduces blood flow to the bladder. A decrease in bladder blood flow accompanies decreased oxygenation of the bladder [2–6]. Proliferation of cells, cellular hypertrophy, and extracellular matrix aggregation are the manifestations of obstructive stress on the bladder. When the bladder is subjected to prolonged stress that cannot manage, it experiences decompensatory response [7,8].

Repeated detrusor ischemia/reperfusion results in the generation of reactive free radicals and the activation of specific phospholipases and proteases that mediate the observed cellular damage. This is the etiology for progressive bladder decompensation [9]. BOO causes an increase in bladder wall thickness and mass that stabilizes and compensates for the demands of bladder emptying against the increased urethral resistance [10,11]. As the bladder wall thickens, cyclical ischemia/reperfusion begins to occur with each micturition cycle [5,12].

If this phase of BOO is prolonged, it induces ischemia of the bladder [5]. Ischemia arises when the bladder becomes over-distended for a long time. This is considered a key determinant of apoptosis. There are still gaps in our knowledge about the underlying signaling mechanisms, despite the range of triggers and mediators suggested that underpin bladder apoptosis [13]. Programmed cell death triggers alterations in the growth equilibrium underpinned by cell proliferation and hypertrophy. Apoptosis is controlled by a sophisticated biochemical mechanism, while its activation is crucially based on an extrinsic and an intrinsic pathway, which triggers apoptosis by activating caspases [14]. The secretion of caspase-activating proteins by mitochondria is essential for the intrinsic pathway [15], which is modulated by two genes with activities promoting and suppressing apoptosis, namely, Bax and Bcl-X. The Bcl-X belongs to the extensive Bcl-2 protein family [16]. These include proteins that are both conducive to and unconducive to apoptosis. This creates a system of homodimers and heterodimers. The relative ratio of apoptosis-promoting proteins to apoptosis-suppressive proteins dictates how sensitive cells are to apoptotic stimuli [14].

This study was undertaken to determine if long-term BOO could induce apoptosis activities and to obtain an expression profile of apoptosis-related genes.

## Materials and methods

### 1) Experimental model of BOO

The Institutional Animal Care and Use Committee of the researcher’s hospital endorsed the experimental protocols (Soonchunhyang university, Seoul hospital, IRB no:2019–4), which we performed in compliance with the National Institute of Health Guide for the Care and Use of Laboratory Animals. The experimental work employed 40 Sprague-Dawley six-week-old female rats weighing 200 g. We divided them into two groups, with the first group representing sham intervention (group 1) and the second group representing eight-week BOO (group 2).

The rats were anesthetized with isoflurane (BKPharm, Goyang, Korea), and we initiated the BOO procedure. We made an incision in the lower abdominal skin to perform urethral dissection. We surrounded the urethra with 4–0 silk sutures, together with a 1-mm diameter metal rod in the extraluminal position. We took out the rod once we had tied off the sutures and had closed the abdominal wall [17]. Generally, within 30 minutes after being returned to their cage, the animal exhibits responsiveness and normal respiration. Lethargy, swelling at incision site, hemorrhage, impaired mobility, aggression, anorexia, signs of pain, infection was monitored (every day until removal of sutures). We assessed pain scores and provided analgesia per the pain score and provided analgesia for first 48 hours per protocol regardless of pain score after surgery. And we monitored at least once daily for signs of pain, distress, incision problems and other signs identified at least until sutures are removed. We performed daily intraperitoneal injections of 10-mg/kg Flomoxef (Ildong, Seoul, Korea) to avoid infection [17].

### 2) Measurement of voiding function

We performed evaluation of the voiding response at eight weeks after BOO procedure, as our previous study [17]. We used isoflurane to anesthetize the Sprague-Dawley rats so that a catheter could be surgically introduced into the bladder. This involved making a midline abdominal incision to expose the bladder, followed by the introduction of a polyethylene (PE) 50 catheter via a small incision in the bladder dome. We subjected the bladder end of the catheter to heating to form a collar, around which we tightened a suture. The other catheter end went through the subcutaneous tissue and came out of the skin. We sutured the muscle and skin separately to close the abdominal incision. The rats reserved for anesthesia-free examination were kept in a restraining cage for 5–6 hours, which allowed time for them to recover from the isoflurane anesthetic. A T-stopcock was linked from the bladder catheter to a pump for ongoing physiological saline administration and to serve as a pressure transducer. We induced recurring voiding responses by administering physiological saline to the bladder at a fixed 0.04-mL/min rate and at an ambient temperature. We investigated the triggered micturition reflexes on restrained rats that had been subjected to urethane anesthesia. We performed investigations of the micturition-inducing intravesical pressure, the pressure threshold (PT), maximal voiding pressure (MVP), and inter contractile interval (ICI) representing the duration between voids or reflex bladder contractions.

### 3) Histology and immunohistochemistry

When we had completed the cystometric assessment, we subjected five rats from each group to cardiac perfusion with 100-mL cold saline and 100-mL 4% paraformaldehyde in PBS under urethan anesthesia as our previous study [17]. We subsequently removed the bladders, fixing them in 4% paraformaldehyde for two days and subjected them to cryoprotection in 30% sucrose for three days. We then used a cryostat (Leica CM 1900) to create 10-μm bladder segments, which underwent staining. We processed the immunofluorescence assessment of fluorescent staining, and we used an Olympus laser confocal fluorescence microscope to visualize the stained sections. We carried out immunostaining for caspase 3 (CAS3) and terminal deoxynucleotidyl transferase dUTP nick end labeling (TUNEL). Then, we heated a 9-mM sodium citrate (pH 5.0) for ten minutes to enable extraction of the antigen for caspase 3 and TUNEL. We suppressed the endogenous peroxidase activity with peroxidase blocking reagent (Dako, Carpinteria, CA). We subjected sections to overnight incubation with primary antibody for TUNEL TACS^®^ 2 TdT-Fluor In Situ Apoptosis Detection Kit (Trevigen Inc, Gaithersburg, MD) and caspase 3 (1:200 dilution, Santa Cruz Biotechnology, Dallas, TX) in a humidified chamber at 4°C. This was followed by overnight incubation of tissue sections as free-floating sections in a mixed primary antibody solution at 4°C. The sections were afterwards incubated for 60 minutes in a mixed secondary antibody solution of Alexa Fluor 488- conjugated anti-mouse IgG (1:200 dilution, Molecular Probe, Eugene, OR) and Alexa Fluor 594-conjugated anti-rabbit IgG (1:200 dilution, Molecular Probe, Eugene, OR) at ambient temperature. Apart from the exclusion of primary antibodies, the preparation of negative control sections was achieved in the same way. An Olympus laser confocal fluorescence microscope permitted observation of the stained sections. Quantitative analysis of histologic examinations was processed with an image analyzer system (National Institutes of Health [NIH] Image J 1.34, https://imagej.nih.gov/ij/index.html).

### 4) mRNA expression

At the end of the cystometric measurement during anesthesia with isoflurane, the animals were euthanized to enable bladder removal as our previous study [17]. We weighed the bladder following removal of surrounding tissue. The next steps were bladder freezing and storage in liquid nitrogen until required for biochemical and molecular biological assays.

We performed extraction of total RNA from cells and samples with Trizol^®^ reagent (Invitrogen, Carlsbad, CA, USA), followed by real-time PCR. We used the threshold cycle value for quantification of the relative gene expression, while the housekeeping geneβ-actin facilitated normalization of relative gene expression. The data represented the mean of the three experiments. The quantitative PCR procedure involved a 10-second cycle at 95°C, 40 cycles for five seconds at 95°C, and a half-minute cycle at 60°C. We performed melting curve analysis (60–95°C) at the end.

### 5) Apoptosis gene real-time PCR array

We used the RNeasy Mini Kit (QIAGEN, Hilden, Germany) in keeping with the manufacturer’s guidelines for isolation and extraction of total RNA as our previous study [17]. We addressed DNA contamination through RNase-free DNase (QIAGEN, Hilden, Germany) treatment of the solution with extracted RNA. We used 2-μg total RNA and the RT2 First Strand Kit (QIAGEN, Hilden, Germany) to conduct the first strand cDNA synthesis in line with the manufacturer’s guidelines. We mixed the first strand cDNA with RT2 qPCR Master Mixes, and aliquoted the mixture into the 96-Well RT2 Profiler PCR Array for rat hypoxia (QIAGEN, Hilden, Germany).

A 96-well plate encompassed SYBR green-optimized primer assays for a comprehensively investigated panel of pertinent and pathway-oriented genes. It comprised 84 genes established to participate in hypoxic response, cell differentiation and metabolism, and 12 sequences for regulation of loading and cDNA quality. In the process of real-time PCR, we heated the plate for ten minutes at 95°C. Subsequently, we performed 40 cycles for 15 seconds at 95°C, and we performed one 60-second cycle at 60°C. We used genomic DNA, reverse transcription, and positive PCR controls for quality regulation, and we used the housekeeping gene β-actin for data normalization. Representation of gene expression alterations took the form of fold rise or reduction. Alterations of more than two-fold constituted the cut-off determining expression. Genes were thought to display upregulation or downregulation if they satisfied the two criteria above.

Table 1 shows primer for real-time PCR of apoptosis, mRNA expression of bladder.

**Table 1:** Primer for real-time PCR.

Gene	Forward	Reverse
Collagen1	TACAGCACGCTTGTGGATGG	CAGATTGGGATGGAGGGAGTT
TGF1	CTCCCGTGGCTTCTAGTGC	GCCTTAGTTTGGACAGGATCTG
GAPDH	AGGTCGGTGTGAACGGATTTG	TGTAGACCATGTAGTTGAGGTCA

### 6) Network analysis

We used network data integration software, Cytoscape version 3.7.2 and stringApp version 1.5.1 for integrating biomolecular interaction networks and states.

### 7) Statistical analysis

Expression of data took the form of mean ± SE. Inter-group comparison of data was based on statistical analysis undertaken via the non-parametric Mann-Whitney U-test. Discrepancies of statistical significance were reflected by a p-value of less than 0.05.

## Results

### 1) Body and bladder weight

**Fig 1:**
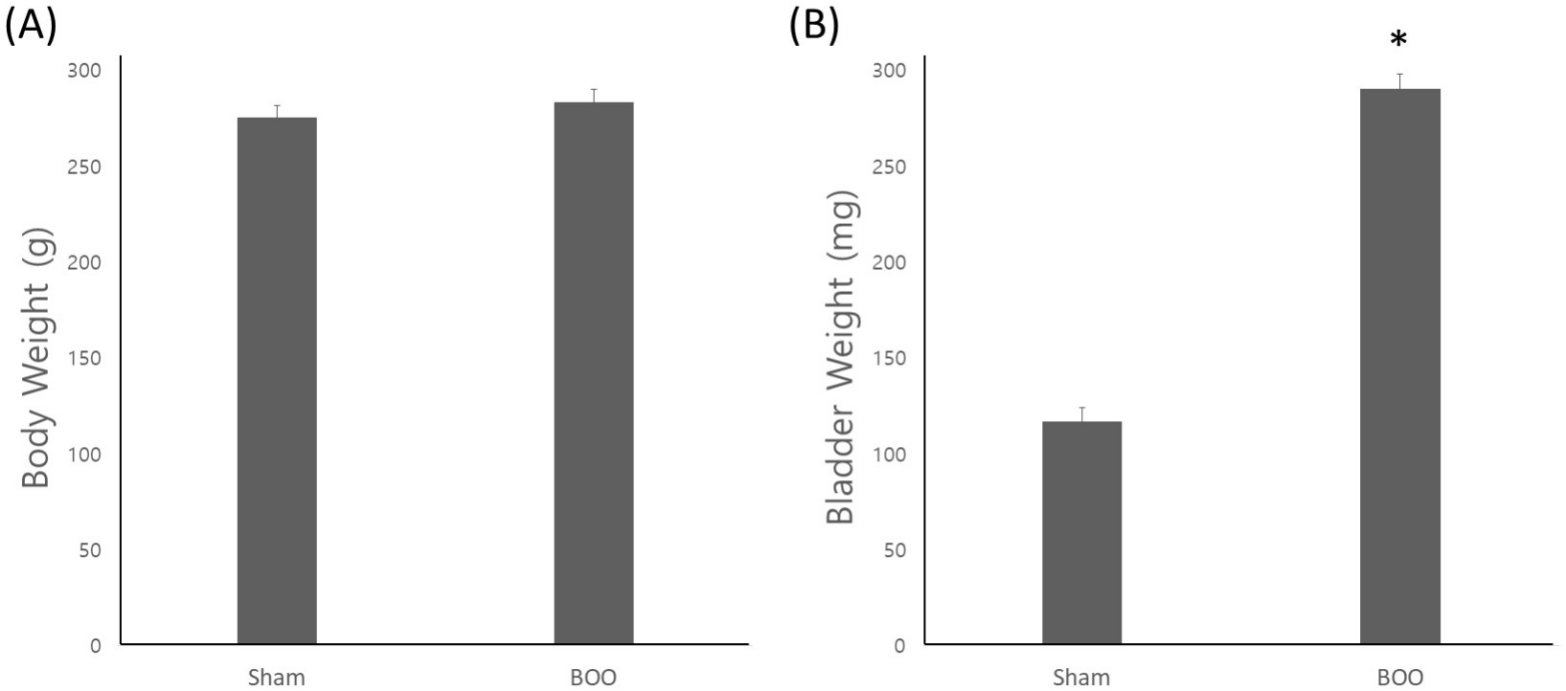
Change of body and bladder weight after operation. There was no significant difference of body weight between groups. The group 2 showed increased bladder weight than group 1 (*p* < 0.05). Sham = sham operation, BOO = 8 weeks after bladder outlet obstruction.

The groups did not differ significantly in terms of body weight. As shown in Fig 1, group 1 had a mean body weight of 275.0 ± 6.3 g, while group 2 had a mean body weight of 283.0 ± 7.0 g. Regarding bladder weight, group 1 had a mean bladder weight of 116.7 ± 7.1 mg, while group 2 had a mean bladder weight of 290.0 ± 7.8 mg. Compared to the sham intervention, group 2 had a greater bladder weight.

### 2) Impaired bladder contractility

**Fig 2:**
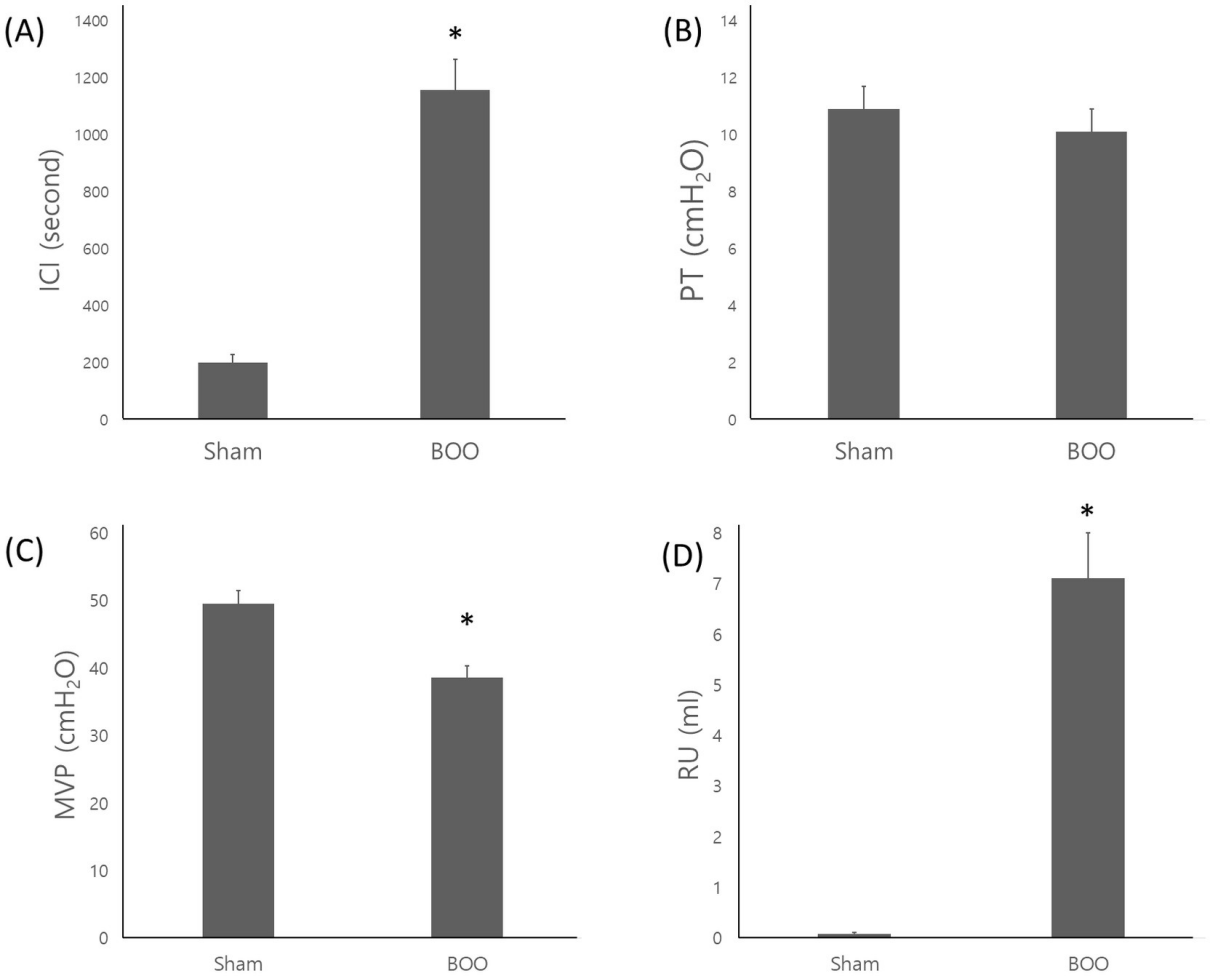
Cystometric parameters after operation. A: Inter-contraction interval (ICI). B: Maximal voiding pressure (MVP). C: Threshold pressure (PT). Sham = sham operation, BOO = 8 weeks after bladder outlet obstruction, Data = mean±standard error. ICI (second) was 200±57, 1156±221 in group 1 and 2. ICI increased after BOO (*p*<0.05). PT (cmH_2_O) was 10.9±0.8, 10.1±0.8 in group 1 and 2. PT had no change among groups. MVP (cmH_2_O) was 49.5±2.1, 38.6±3.5 in group 1 and 2. RU (ml) was 0.08±0.06, 7.1±1.8 in group 1 and 2. MVP decreased and RU increased in group 2.

The ICI was 200 ± 57 second, 1156 ± 221 second in group 1 and 2. ICI increased after BOO formation (*p* < 0.05). PT was 10.9 ± 0.8 cmH_2_O, 10.1 ± 0.8 cmH_2_O in group 1 and 2 (*p* > 0.05). PT had no change between groups. MVP was 49.5 ± 2.1 cmH_2_O, 38.6 ± 3.5 cmH_2_O in group 1 and 2 (*p* < 0.05). Post-void residual urine volume (RU) was 0.08 ± 0.06 mL, 7.1 ± 1.8 mL in group 1 and 2 (*p* < 0.05). ICI increased, MVP decreased and RU increased in group 2 (Fig 2).

### 3) mRNA expression of bladder in the group of BOO

**Fig 3:**
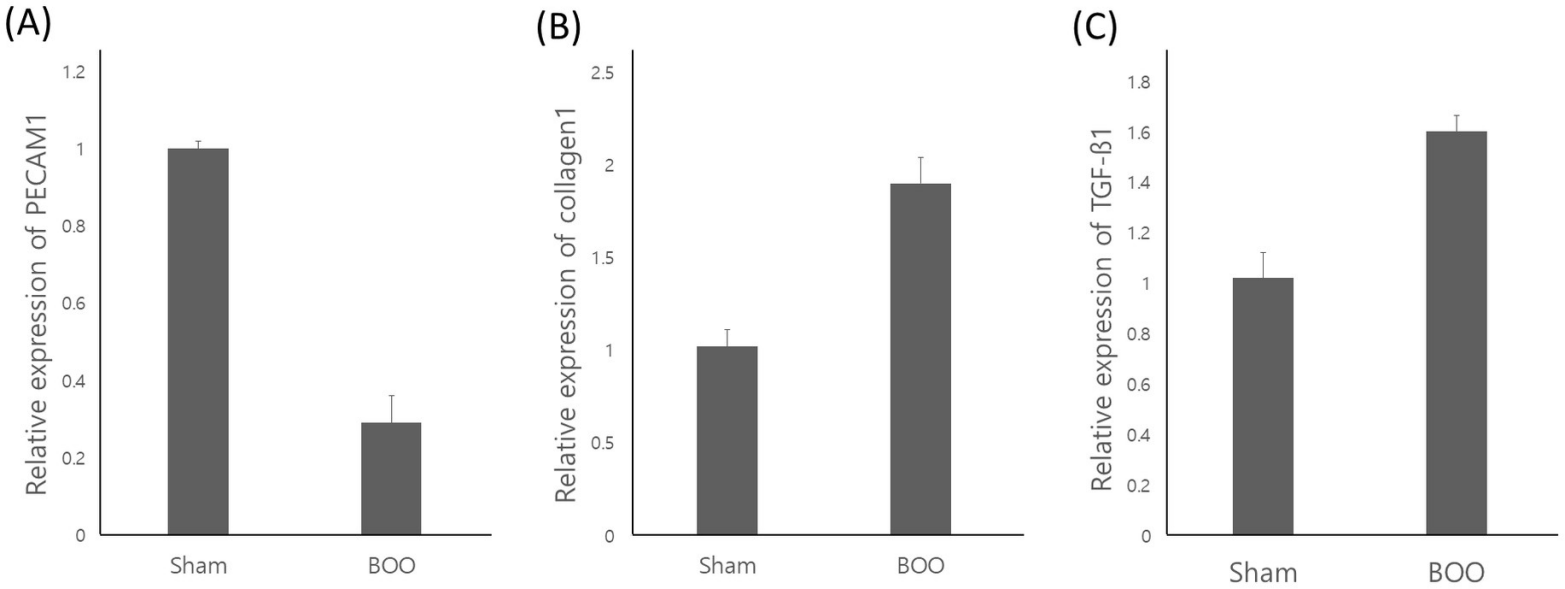
mRNA expression of bladder in group 2. mRNA expression of PECAM1 decreased, collagen1and TGF-beta1 increased in group 2 (*p*<0.05). Sham = sham operation, BOO = 8 weeks after bladder outlet obstruction.

The mRNA expression of Platelet and Endothelial Cell Adhesion Molecule (PECAM) 1 decreased in group 2. A decrease in PECAM1 immunostaining was interpreted as a decrease in the number of blood vessels and a decrease in blood supply. The mRNA expression of collagen-1 and TGFb-1 increased in group 2 (*p* < 0.05) (Fig 3).

### 4) Histopathology

**Fig 4:**
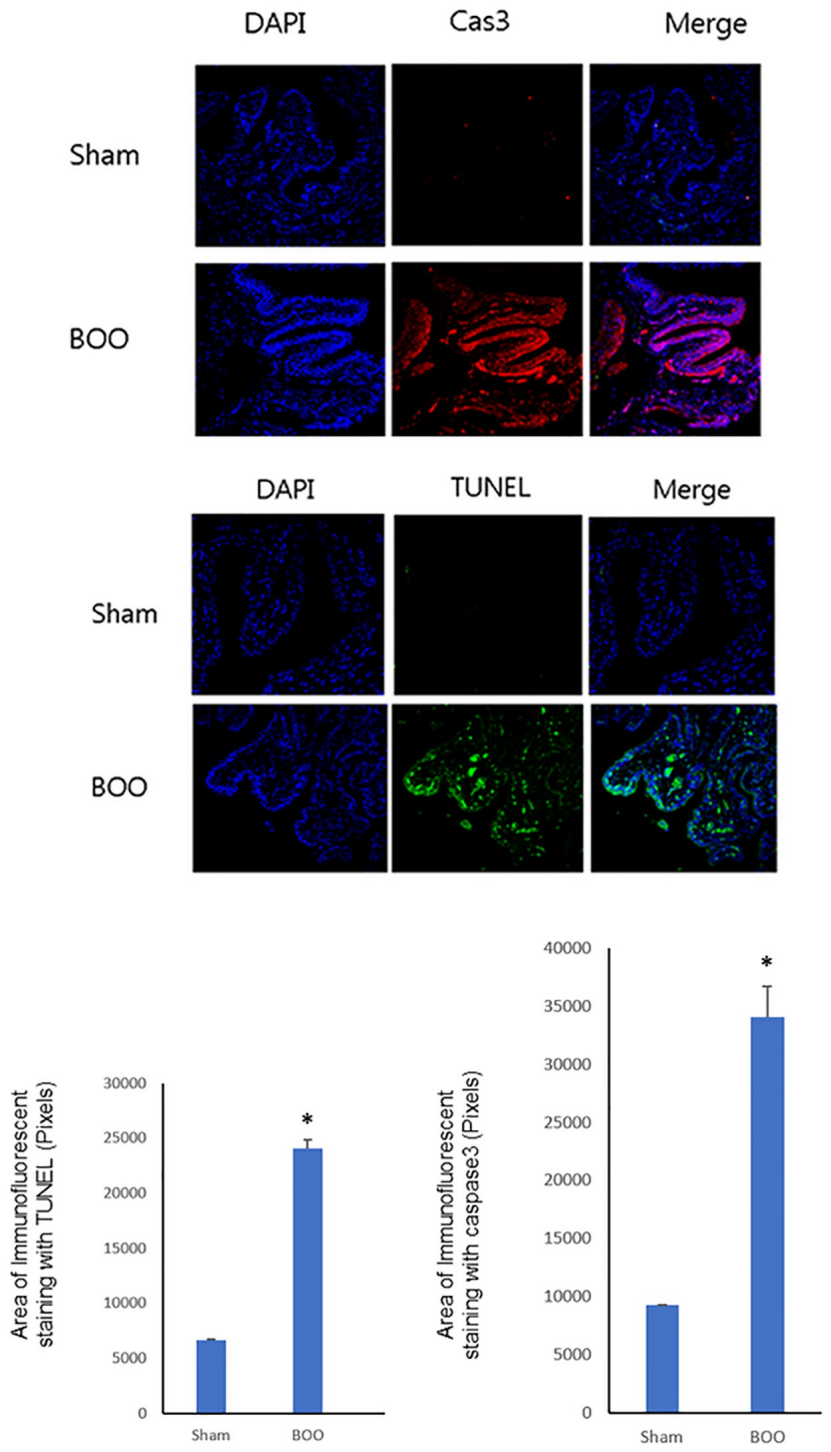
Immunofluorescent staining of bladder tissues. Staining with CAS3, TUNEL demonstrated increased CAS3, TUNEL positive apoptosis in group 2.

Immunofluorescent staining with CAS3, TUNEL, as an indicator of apoptosis, showed increased CAS3, TUNEL positive cells in group 2 (*p* < 0.05) (Fig 4).

### 5) Apoptosis signaling pathway PCR array

**Fig 5:**
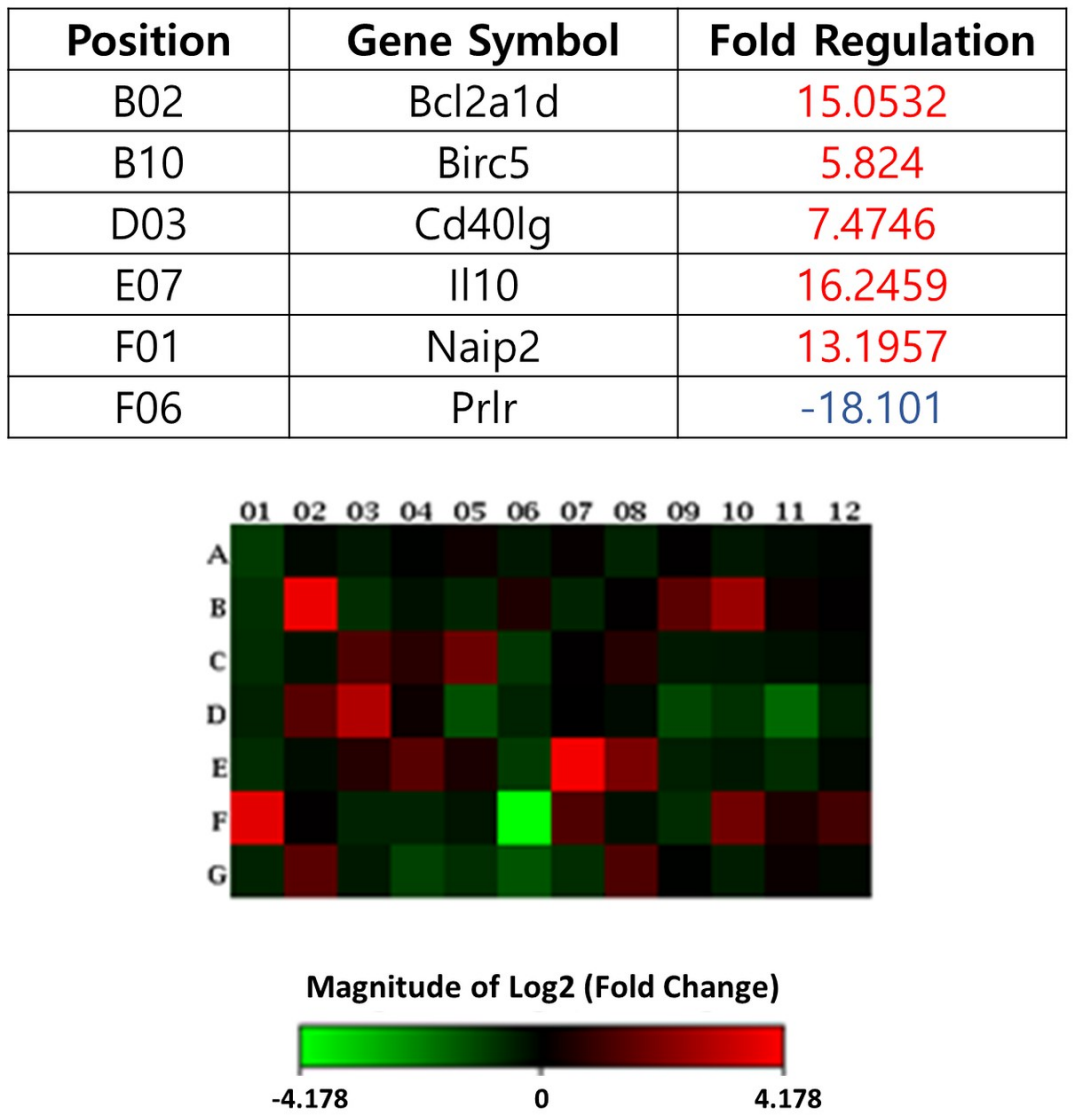
Gene expression profiling of rat bladder under apoptosis conditions. The interactive network during hypoxia. An RT2 Profiler^™^ PCR Array was used to screen a panel of 84 genes associated with rat hypoxia in group 1 and 2. 5 genes were 2-fold up-regulated, and 1 gene was at least 2-fold down-regulated in group 2, compared with group 1. Changes were confirmed by Heat mapping.

S1 Table shows the genes of RT2 Profiler PCR array for apoptosis. From 84 apoptosis genes, five genes associated with Bcl2a1d (15.1), Birc5 (5.8), Cd40lg (7.5), Il10 (16.2), Naip2 (13.2) exhibited upregulation of at least two-fold, while one gene associated with PRLR (-18.1) exhibited at least a two-fold downregulation in group 2 by comparison to group 1. Among the genes with action against apoptosis were Bcl2a1d, Birc5, Cd40lg, Il10, Naip2 and PRLR. The Birc5 and Cd40lg were respectively caspase suppressor and negative regulator. We confirmed changes by Heat mapping (Fig 5).

### 6) Network analysis

**Fig 6:**
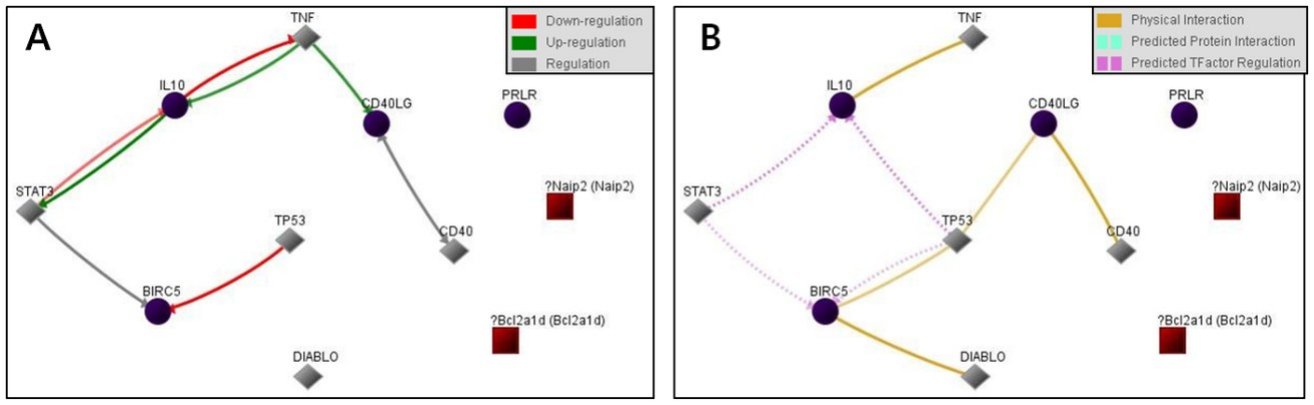
Gene expression profiling of rat bladder under apoptosis conditions. The interactive network during apoptosis. Relationship between selected genes showed that genes were influenced each other through TNF, STAT3 and TP53 (Cytoscape string app. A: Regulation of expression network, B: Interaction network).

The relationship between selected genes showed TNF, STAT3, and TP53 mediated reciprocal influence among the genes (Fig 6).

## Discussion

In our study, the up-regulation of 5 genes: Bcl-2(15.1), Birc5(5.8), Cd40lg(7.5), Il10(16.2) and Naip2(13.2) was confirmed in the BOO group, while one gene associated with PRLR was down-regulated. Cd40lg, Il10, Naip2 and PRLR are genes involved in apoptosis. Bcl-2 inhibits and Bax accelerates apoptosis. However, the relative ratios of apoptosis-promoting proteins (such as Bax) to apoptosis-suppressing proteins (Bcl-2) is important. The relative ratios of upregulated Bcl2a1d, Birc5, Cd40lg, Il10, Naip2 and downregulated gene of PRLR dictate how sensitive bladder cells are to apoptotic stimuli. So, it is thought that the change in the ratio of proapoptotic and antiapoptotic genes is more important than the change of a single gene.

We created a model of long term bladder outlet obstruction in the same way as in our previous study [17]. This prolonged bladder outlet obstruction is the etiology of progressive bladder failure. BOO thickens the bladder wall to stabilize and compensate for the evacuation requirement for increased urethral resistance, thereby increasing bladder mass [10,11]. When the urethral pressure rises during BOO, the intravesical pressure increases and the bladder wall thickens. This causes ischemia of the bladder wall, which can lead to OAB if it is repeated [18]. As the bladder wall thickens, cyclical ischemia/reperfusion begins to occur with each micturition cycle [5,12]. In this study, we found increased bladder weight and thickened bladder in the BOO group (Fig 1). We also found decreased bladder contractility that involves decompensated bladder (Fig 2). This resulted in decreased expression of PECAM1 mRNA, a marker of vascular endothelial cells, in the BOO group (Fig 3). Usually PT is the minimum bladder pressure for urination. But we used PT as the pressure for zero cystometry adjustment during bladder storage and contraction. The low MVP suggested decreased contractility in the decompensated phase. However, we have not checked the serial change of MVP as time flows, so we do not know how long compensated phase was. We do not know the normal ICI reference in rats. Therefore, the sham operation group was considered as a control group and the study was conducted.

Empirical work on adult rabbits revealed that BOO engendered bladder hypertrophy and intensification of apoptosis that showed increased nuclear DNA fragmentation associated with apoptosis and increased expression of TGFb1 mRNA during decompensated [19]. Proliferation of fibroblasts, regulation of matrix component synthesis, and stimulation of apoptosis of smooth muscle cells are all underpinned by the multifunctional cytokine TGF β1, which in turn is controlled by bladder distension [20]. Also in this study, eight weeks of BOO led to an increase of TGFb1 mRNA activity in the bladders (Fig 3).

The activation of caspase, important enzymes in apoptosis, is induced by specific cell-surface receptors (e.g. Fas ligand stimulation), mitochondrial cascade (e.g. irradiation) or other promoters. Caspase-3 is found at the convergence of the Fas ligand stimulation and the mitochondrial cascade. The activated molecule can trigger apoptosis by undertaking the cleavage of many structural proteins, cellular enzymes, and other cellular substrates [21]. We were able to confirm that caspase-3 activity was increased in the bladder of BOO rats (Fig 4).

Apoptosis is a suggested mechanism in obstructed bladder. Apoptosis may be triggered in BOO by the endoplasmic reticulum stress induced by bladder hypoxic stress [22]. One study conducted on rabbits reported that partial BOO engendered focal zones of moderate-to-severe hypoxia in the detrusor smooth muscle and subserosal areas. This, at the same time as expansion in bladder size and reduced smooth muscle contraction and selective metabolic dysfunction, engendered an equivalent-to-intermediate decompensation phase [23]. TUNEL staining of the BOO bladder showed that significantly higher apoptosis counts in bladders with BOO. In this study, we found apoptosis in the decompensated bladder, using TUNEL immunostaining (Fig 4). The significant increase in apoptotic smooth muscle cells occurred within the detrusor muscle bundles.

We conducted an apoptosis real-time PCR array to investigate the molecular mechanism of bladder compensation. This revealed that five genes displayed at least a two-fold upregulation while one gene displayed at least a two-fold downregulation in the BOO group compared with the sham group. In this study, the upregulated genes in the BOO group belong to Bcl2a1d, Birc5, Cd40lg, Il10, Naip2 and we found the downregulated genes belonging to PRLR. (Fig 5). The relative ratios of apoptosis-promoting proteins to apoptosis-suppressing proteins which dictate how sensitive bladder cells are to apoptotic stimuli include the upregulated genes of Bcl2a1d, Birc5, Cd40lg, Il10, and Naip2 and downregulated gene of PRLR.

Bcla1d (B-cell leukemia lymphoma 2 related protein A1d), is a member of the Bcl-2 family. The secretion of caspase-activating proteins is essential for the intrinsic pathway [15], which is modulated by two genes with activities promoting and suppressing apoptosis, namely, Bax and Bcl-X. Bcl-2 inhibits and Bax accelerates apoptosis [24]. In this study, a heatmap showed no significant change in Bax, and apoptosis gene real-time PCR array showed a 15.1-fold upregulation of Bcl2a1d. Therefore, the ratio of bladder expression of Bcl-2 to Bax increased. In the decompensated bladder compared to the sham operated bladder, apoptosis is inhibited in response to obstruction of the antiapoptosis gene Bcl-2 increasing with obstruction.

The Birc5 (Baculoviral IAP repeat-containing 5) is a member of apoptosis family inhibitors. An apoptosis gene real-time PCR array demonstrated a 5.8-fold upregulation. This functions to inhibit caspase activation and leads to negative regulation of apoptosis [25]. In the decompensated bladder, apoptosis is inhibited in response to obstruction with antiapoptosis gene Birc5, increasing with obstruction.

Cd40lg (CD40 ligand) is a member of the TNF superfamily of molecules and binds to CD40 antigen presenting cells (APC), which lead to apoptosis. Induction or suppression of apoptosis by Cd40lg has been reported [26,27]. Apoptosis gene real-time PCR array demonstrated a 5.8-fold upregulation. Apoptosis is induced or inhibited in response to obstruction with antiapoptosis gene Cd40lg increasing with obstruction, in decompensated bladder.

Il10 (Interleukin 10) modulates the induction of apoptosis with TNF-α. Interleukin-10 induces macrophage apoptosis [28]. The apoptosis gene real-time PCR array demonstrated a 16.2-fold upregulation of Il10. In the decompensated bladder, it is suggested that apoptosis is induced in response to obstruction, with the antiapoptosis gene Il10 increasing with obstruction. Naip2 (NLR family, apoptosis inhibitory protein 2) is the NLR (NOD-like receptor) family and inhibits apoptosis [29]. In this study, the apoptosis gene real-time PCR array demonstrated a 13.2-fold upregulation of Naip2. Apoptosis is induced in response to obstruction, accompanied by antiapoptosis gene Naip2 increasing with obstruction.

Prolactin stimulates cells to proliferate and extends cell life, thus regulating immune system functions. With expression in all immune cells and belonging to the superfamily of hematopoietin cytokine receptors, the prolactin receptor (PRL-R) underpins the activities of prolactin [30–32]. PRL can also suppress apoptosis in a range of types of cells, such as the Nb2 rat lymphoma cell line, although this property has not been widely addressed [33,34]. In this study, apoptosis gene real-time PCR array demonstrated PRLR (Prolactin receptor) (-18.1). It means positive regulation of apoptosis. In the decompensated bladder, it is suggested that apoptosis is induced in response to obstruction with antiapoptosis gene PRLR increasing with obstruction.

A gene regulatory network defines the biological interplay between genes, which also offers insight into mechanisms of cell signaling and regulation. This network indicates the manner of interaction between a series of genes to create a functional module and the nature of the correlation between various gene modules. The gene regulatory network correlates a network topology without scale to a couple of hub genes with close connection and a large number of nodes with poor connection [35]. Dominating the gene regulatory network, the hub genes are typically vital in biological systems. Possible clinical uses may be discovered from research on the gene regulatory network, as this can mediate methodical gene functional annotation [36] and distinguish the hub genes [37]. The present work established that TNF, STAT3 and TP53 mediated the reciprocal influence among genes (Fig 6). This is similar to the results of other previous studies. In a study by Koeck et al., TNF-treated bladder smooth muscle cells revealed similarities with human BOO, and reported that TNF was associated with the progression of BOO [38]. Increased expression of STAT3 in BOO was also confirmed, and association with TP53 was recently reported [17,39].

Expression analysis with real-time PCR array has its usefulness, yet it is not without shortcomings. Bladder tissue consists primarily of smooth muscle, but the extracted bladder samples contained a variety of cells. This might have contributed to the differential gene expression observed in the present work. Furthermore, we found that the gene modifications and the BOO-related bladder dysfunction were correlated. Additional empirical studies must be conducted to determine whether genes with differential expression mediate, indicate, or are irrelevant to BOO-related impaired bladder function. Many PCR array analyses are affected by this issue, and therefore the current work does not go beyond the formulation of hypotheses.

The present investigation lacks completeness because of the employed RT2 Profiler PCR Array for rat hypoxia (QIAGEN, Hilden, Germany). It consists solely of probe sets for more than 84 genes and transcripts, including established genes of rat apoptosis. But it does not probe sets for every rat apoptosis gene. Moreover, one can overlook key alterations happening at an earlier or later point in BOO-related bladder dysfunction progression if gene expression is analyzed at just one time point. To identify the genes with direct involvement in bladder dysfunction from BOO, it may be useful to conduct a more inclusive examination of temporal alterations, according to expression patterns over time.

## Conclusions

The changes of proapoptotic and antiapoptotic genes determine the sensitivity of the bladder cells to apoptotic stimuli, including the upregulated Bcl2a1d, Birc5, Cd40lg, Il10, Naip2 genes and the downregulated PRLR gene in impaired contractility and long-term BOO causes. The ratio of proapoptotic and antiapoptotic genes is more important than the change of a single gene. Although we cannot confirm whether this finding is the result of the decompensated phase of the bladder or the process, the gene expression profiles could explain molecular mechanisms of apoptosis in impaired contractility due to long term BOO with further studies. In the future, this understanding will be helpful in treating BOO patients.

## Supporting information

S1 Table Genes of RT2 Profiler PCR array. (DOCX)

S1 Raw data (ZIP)
