# Supplementary material for: Changes of proapoptotic and antiapoptotic genes affect sensitivity to apoptotic stimuli in impaired contractility due to long term bladder outlet obstruction
Source: PLoS One. 2022 Dec 27;17(12):e0279503. doi: 10.1371/journal.pone.0279503 (PMC9794071; doi:10.1371/journal.pone.0279503)
Supplement: S1 Table — (DOCX) [file pone.0279503.s001.docx]

Supplementary table 1. Genes of RT2 Profiler PCR array

| Position | UniGene | GenBank | Symbol | Description |
| --- | --- | --- | --- | --- |
| A01 | Rn.3105 | NM_001100850 | Abl1 | C-abl oncogene 1, receptor tyrosin kinase |
| A02 | Rn.203165 | NM_031356 | Aifm1 | Apoptosis-inducing factor, mitochondrion-associated 1 |
| A03 | Rn.11422 | NM_033230 | Akt1 | V-맛 murine thymoma viral oncogene homolog 1 |
| A04 | Rn.3318 | NM_013132 | Anxa5 | Annexin A5 |
| A05 | Rn.64522 | NM_023979 | Apaf | Apoptotic peptidase activating factor 1 |
| A06 | Rn.103083 | NM_001127379 | Api5 | Apoptosis inhibitor 5 |
| A07 | Rn.145049 | NM_001107757 | Aven | Apoptosis, caspase activation inhibitor |
| A08 | Rn.36696 | NM_022698 | Bad | BCL2-associated agonist of cell death |
| A09 | Rn.16320 | NM_001106647 | Bag1 | BCL2-associated athanogene |
| A10 | Rn.14598 | NM_053812 | Bak1 | BCL2-antagonist/killer 1 |
| A11 | Rn.10668 | NM_017059 | Bax | Bcl2-assocated X protein |
| A12 | Rn.13007 | NM_031328 | Bcl10 | B-cll CLL/lymphoma 10 |
| B01 | Rn.9996 | NM_016993 | Bcl2 | B-cll CLL/lymphoma 2 |
| B02 | Rn.19770 | NM_133416 | Bcl2a1d | B-cell leukemia/lymphoma 2 related protein A1d |
| B03 | Rn.10323 | NM_031535 | Bc12l1 | Bcl2-like 1 |
| B04 | Rn.82709 | NM_022612 | Bcl2l11 | BCL2-like 11 (apoptosis facilitator) |
| B05 | Rn.44267 | NM_021850 | Bcl2l2 | Bcl2-like 2 |
| B06 | Rn.31142 | NM_022684 | Bid | BH3 interacting domain death agonist |
| B07 | Rn.38487 | NM_053704 | Bik | BCL2-interacting killer (apoptosis-inducing) |
| B08 | Rn.205955 | NM_021752 | Birc2 | Baculoviral IAP repeat-containing 2 |
| B09 | Rn.64578 | NM_023987 | Birc3 | Baculoviral IAP repeat-containing 3 |
| B10 | Rn.54471 | NM_022274 | Birc5 | Baculoviral IAP repeat-containing 5 |
| B11 | Rn.11821 | NM_001106835 | Bnip2 | BCL2/adenovirus E1B interacting protein 2 |
| B12 | Rn.2060 | NM_053420 | Bnip3 | BCL2/adenovirus E1B interacting protein 3 |
| C01 | Rn.44461 | NM_017312 | Bok | BCL2-related ovarian killer |
| C02 | Rn.138066 | NM_001130554 | Card10 | Caspase recruitment domain family, member 10 |
| C03 | Rn.37508 | NM_012762 | Casp1 | Caspase 1 |
| C04 | Rn.81078 | NM_130422 | Casp12 | Caspase 12 |
| C05 | Rn.198773 | XM_234878 | Casp14 | Caspase 14 |
| C06 | Rn.1438 | NM_022522 | Casp2 | Caspase 2 |
| C07 | Rn.10562 | NM_012922 | Casp3 | Caspase 3 |
| C08 | Rn.16195 | NM_053736 | Casp4 | Caspase 4, apoptosis-related cysteine peptidase |
| C09 | Rn.88160 | NM_031775 | Casp6 | Caspase 6 |
| C10 | Rn.53995 | NM_022260 | Casp7 | Caspase 7 |
| C11 | Rn.54474 | NM_022277 | Casp8 | Caspase 8 |
| C12 | Rn.198715 | NM_001107921 | Cas98ap2 | Caspase 8 associated protein 2 |
| D01 | Rn.32199 | NM_031632 | Casp9 | Caspase 9, apoptosis-related cysteine peptidase |
| D02 | Rn.25180 | NM_134360 | Cd40 | CD40 molecule, TNF receptor superfamily member 5 |
| D03 | Rn.44218 | NM_053353 | Cd40lg | CD40 ligand |
| D04 | Rn.204752 | NM_057138 | Cflar | CASP8 and FADD-like apoptosis regulator |
| D05 | Rn.8171 | NM_001170467 | Cidea | Cell death-inducing DFFA-like effector a |
| D06 | Rn.204016 | NM_001108869 | Cideb | Cell death-inducing DFFA-like effector b |
| D07 | Rn.2202 | NM_012839 | Cycs | Cytochrome c, somatic |
| D08 | Rn.7262 | NM_138910 | Dad1 | Defender against cell death 1 |
| D09 | Rn.23108 | NM_001107335 | Dapk1 | Death associated protein kinase 1 |
| D10 | Rn.6514 | NM_053679 | Dffa | DNA fragmentation factor, alpha subunit |
| D11 | Rn.67077 | NM_053362 | Dffb | DNA fragmentation factor, beta polypeptide (caspase-activated DNase) |
| D12 | Rn.9090 | NM_001008292 | Diablo | Diablo homolog (Drosophila) |
| E01 | Rn.16183 | NM_152937 | Fadd | Fas (TNFRSF6)-associated via death domain |
| E02 | Rn.106419 | NM_080895 | Faim | Fas apoptotic inhibitory molecule |
| E03 | Rn.162521 | NM_139194 | Fas | Fas (TNF receptor superfamily, member 6) |
| E04 | Rn.9725 | NM_012908 | Faslg | Fas ligand (TNF superfamily, member 6) |
| E05 | Rn.10250 | NM_024127 | Gadd45a | Growth arrest and DNA-damage0inducible, alpha |
| E06 | Rn.89639 | NM_057130 | Hrk | Harakiri, BCL2 interacting protein (contains only BH3 domain) |
| E07 | Rn.9868 | NM_012854 | Il10 | Interleukin 10 |
| E08 | Rn.160577 | NM_080769 | Lta | Lymphotoxin alpha (TNF superfamily, member 11) |
| E09 | Rn.19329 | NM_001008315 | Ltbr | Lymphotoxin beta receptor (TNFR superfamily, member 3) |
| E10 | Rn.34914 | NM_053842 | Mapk1 | Mitogen-activated protein kinase 1 |
| E11 | Rn.44266 | NM_053777 | Mapk8ip1 | Mitogen-activated protein kinase 8 interacting protein 1 |
| E12 | Rn.129914 | NM_021846 | Cml1 | Myeloid cell leukemia sequence 1 |
| F01 | Rn.92423 | XM_226742 | Naip2 | NLR family, apoptosis inhibitory protein 2 |
| F02 | Rn.2411 | XM_342346 | Nfkb1 | Nuclear factor of kappa light polypeptide gene enhancer in B-cells 1 |
| F03 | Rn.86956 | NM_053516 | Nol3 | Nucleolar protein 3 (apoptosis repressor with CARD domain) |
| F04 | Rn.9346 | NM_017141 | Polb | Polymerase (DNA directed), beta |
| F05 | Rn.2511 | NM_017169 | Prdx2 | Peroxiredoxin 2 |
| F06 | Rn.9757 | NM_012630 | Prlr | Prolactin receptor |
| F07 | Rn.7817 | NM_172322 | Pycard | PYD and CARD domain containing |
| F08 | Rn.102179 | XM_342810 | Ripk2 | Receptor-interacting serine-threonine kinase 2 |
| F09 | Rn.41053 | NM_001012066 | Sphk2 | Sphingosine kinase 2 |
| F10 | Rn.2275 | NM_012675 | Tnf | Tumor necrosis factor (TNF superfamily, member 2) |
| F11 | Rn.105558 | NM_001108873 | Tnfrsf10b | Tumor necrosis factor receptor superfamily, member 10b |
| F12 | Rn.202973 | NM_012870 | Tnfrsf11b | Tumor necrosis factor receptor superfamily, member 11b |
| G01 | Rn.11119 | NM_013091 | Tnfrsf1a | Tumor necrosis factor receptor superfamily, member 1a |
| G02 | Rn.83633 | NM_130426 | Tnfrsf1b | Tumor necrosis factor receptor superfamily, member 1b |
| G03 | Rn.83627 | NM_145681 | Tnfsf10 | Tumor necrosis factor receptor superfamily, member 10 |
| G04 | Rn.3211 | NM_001001513 | Tnfsf12 | Tumor necrosis factor receptor superfamily, member 12 |
| G05 | Rn.54443 | NM_030989 | Tp53 | Tumor protein p53 |
| G06 | Rn.50333 | NM_223012 | Tp53bp2 | Tumor protein p53 binding protein, 2 |
| G07 | Rn.42907 | NM_019221 | Tp63 | Tumor protein p63 |
| G08 | Rn.103860 | NM_001108696 | Tp73 | Tumor protein p73 |
| G09 | Rn.18545 | NM_001100480 | Tradd | TNFRSF1A-associated via death domain |
| G10 | Rn.105232 | NM_001107815 | Traf2 | Tnf receptor-associated factor 2 |
| G11 | Rn.12033 | NM_001108724 | Traf3 | Tnf receptor-associated factor 3 |
| G12 | Rn.91239 | NM_022231 | Xiap | X-linked inhibitor of apoptosis |
| H01 | Rn.94978 | NM_031144 | Actb | Actin, beta |
| H02 | Rn.1868 | NM_012512 | B2m | Beta-2 microglobulin |
| H03 | Rn.47 | NM_012583 | Hprt1 | Hypoxanthine phosphoribosyltransferase 1 |
| H04 | Rn.107896 | NM_017025 | Ldha | Lactate dehydrogenase A |
| H05 | Rn.973 | NM_001007604 | Rplp1 | Ribosomal protein, large, P1 |
| H06 | N/A | U26919 | RGDC | Rat Genomic DNA Contamination |
| H07 | N/A | SA_00104_ | RTC | Reverse Transcription control |
| H08 | N/A | SA_00104 | RTC | Reverse Transcription control |
| H09 | N/A | SA_00104 | RTC | Reverse Transcription control |
